# Nitrogen fixation in two coastal upwelling regions of the Taiwan Strait

**DOI:** 10.1038/s41598-017-18006-5

**Published:** 2017-12-14

**Authors:** Zuozhu Wen, Wenfang Lin, Rong Shen, Haizheng Hong, Shuh-Ji Kao, Dalin Shi

**Affiliations:** State Key Laboratory of Marine Environmental Science, Xiamen University, Xiamen, Fujian 361102 P. R. China

## Abstract

Recent studies have demonstrated that dinitrogen fixation can be important in nutrient-rich coastal upwelling regions. During a cruise to the Taiwan Strait in summer 2015, we found that the nitrogen fixation rate in surface waters ranged from below detection limits to 7.51 nmol N L^−1^ d^−1^. Higher rates accompanied by low N:P ratios (1–10.4:1) associated with low temperatures occurred in the surface water where the Pingtan and the Dongshan upwelling regions met (the NE area). In contrast, insignificant rates were observed in the southwest area of the Dongshan upwelling region (the SW area) with sufficient N and deficient P, and therefore high N:P ratios (e.g., >43 at station C2) due largely to the influence of the Pearl River plume. Diatom-associated symbionts (het-1; 10^4^–10^6^ copies L^−1^) that are efficient in organic matter export were found to dominate the other diazotrophic groups that were surveyed, which may represent a direct relationship between new nitrogen input and export in the upwelling regions. Our results suggest a hydrographical influence on the diazotroph community and N_2_ fixation in coastal upwelling regions.

## Introduction

Marine dinitrogen (N_2_) fixers are able to convert dissolved nitrogen gas into bioavailable nitrogen (N), providing new nitrogen to the photic zone for the net sequestration of atmospheric carbon dioxide (CO_2_)^1,2^. For more than two decades there have been attempts to estimate oceanic N_2_ fixation, but most work has focused on the warm and oligotrophic open ocean waters^3–5^. Recent studies have shown that oceanic regions that are traditionally regarded as unfavorable for N_2_ fixation could also be important^6–14^. For example, nutrient-replete coastal upwelling regions in the tropical and sub-tropical oceans, which play a disproportionately important role in nutrient cycling^15^, have been demonstrated to have significant N_2_ fixation potential^6,8,9,11,12,16^.

It has been shown that active N_2_ fixation may occur in an upwelling region with enriched nutrients [e.g., N, phosphorus (P), silicon (Si) and potentially iron (Fe)] sourced from the subsurface where high rates of water column denitrification are often observed^6,8,11^. In such cases, a high nitrate (NO_3_^−^) concentration does not inhibit N_2_ fixation, and factors such as the N:P ratios of the upwelled water, the level of oxygen deficiency in the water column, and temperature may be responsible for the relatively high levels of N_2_ fixation observed^6,11^. In contrast, in the upwelling region off Vietnam and in the Northwest African upwelling region, high diazotrophic activity has been recorded in areas bordering instead of in the actual upwelling region^9,12^. External factors, such as dust input and river plume intrusion that may provide micronutrients and enhance the stability of the water column, could have promoted N_2_ fixation in such adjacent zones of coastal upwelling regions^9,12^. Unlike the two scenarios described above, Zhang, *et al*. recently proposed a “transition zone” scenario where coastal upwelling regions may induce coupled physical and biological effects, consequently modulating N_2_ fixation^16^. These previous studies therefore suggest that the response of diazotrophs to upwelling events and the underlying mechanisms are not likely uniform across coastal upwelling systems that have different hydrographic and biogeochemical characteristics (e.g., shelf width, bathymetry, benthic environment, seasonal upwelling intensity, and extent of external nutrient inputs), which clearly warrants further investigation.

**Figure 1:**
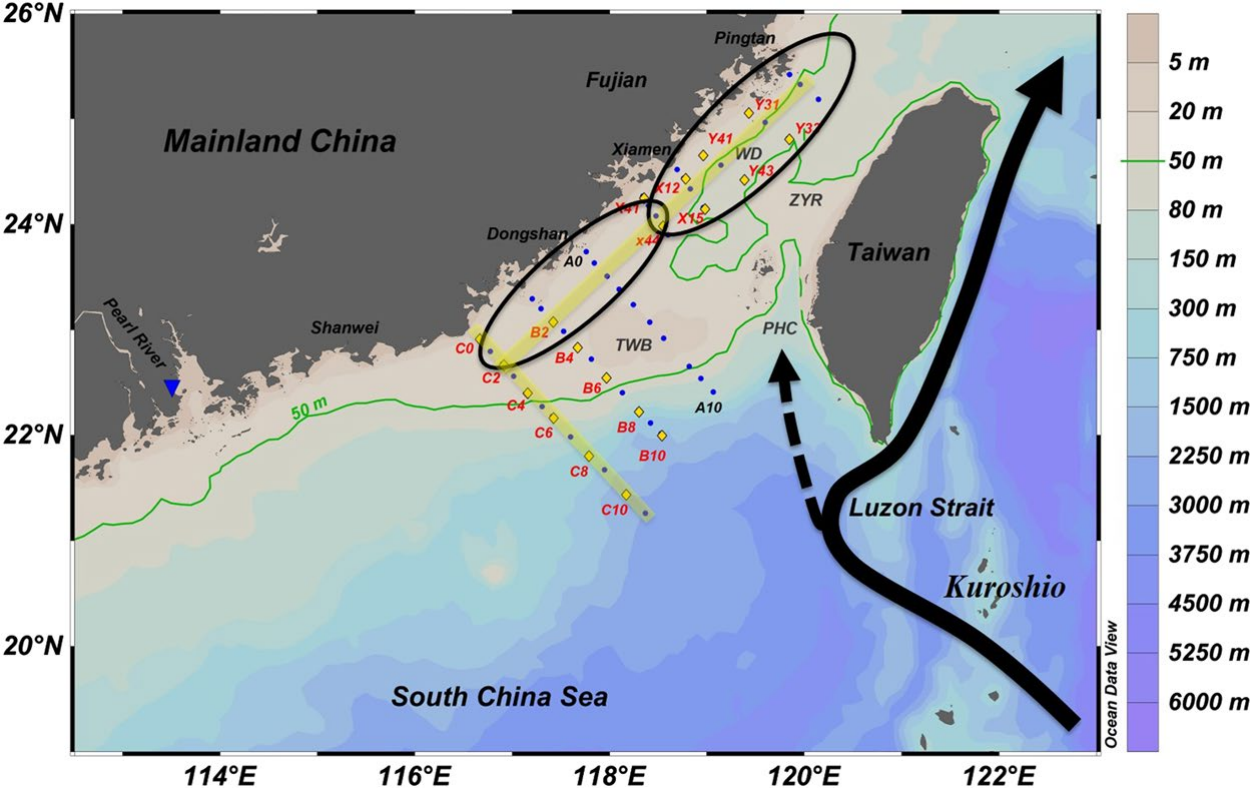
Map of the Taiwan Strait showing the topography and the locations of the sampling stations. The yellow diamonds indicate stations where surface N_2_ fixation rates were assessed, the blue dots indicate those where parameters except N_2_ fixation rate were measured, and the blue triangle indicates the Pearl River outlet. Ellipses indicate the Dongshan Upwelling (DSU) and Pingtan Upwelling (PTU) regions. The black solid line is the Kuroshio Current, and the black dashed line is the Kuroshio branch. The light-yellow lines indicate the two sections demonstrated in Fig. 2. TWB (Taiwan Bank), PHC (Penghu Channel), ZYR (Zhangyun Ridge), and WD (Wuchou Depression). This figure was created using Ocean Data View (Schlitzer, R., Ocean Data View, http://odv.awi.de, 2017).

Located between Taiwan Island and the southeastern Chinese mainland, the Taiwan Strait (TWS) is approximately 180 km wide and 350 km long, with an average depth of 60 m (Fig. 1). Water circulation and the formation of upwelling regions in the TWS are driven by complex bottom topography coupled with strong monsoon forcing^17–22^. Upwelling regions in the TWS have been well defined and studied previously^17,22–25^. Among them, the Dongshan upwelling (DSU) and the Pingtan upwelling (PTU) regions are wind-driven, topographically-forced coastal upwelling systems, which form in the western TWS during the summer monsoon period.

To our knowledge, the spatial distribution of N_2_ fixation and its controlling mechanisms in the coastal upwelling regions of the TWS are essentially unknown^26^. The unique characteristics in or near the DSU and PTU regions are as follows: 1) Compared with other typical coastal upwelling regions, shallower waters and more complex bottom topography in the TWS likely create a more turbulent surface environment that has traditionally been considered unfavorable for the growth of filamentous diazotrophs such as *Trichodesmium* spp.; 2) The connection between offshore Ekman transported coastal water and oceanic water is cut off by Taiwan Island, and thus the “transition zone” described by Zhang, *et al*. is unlikely to be applicable in the TWS^16^; and 3) The eastward flow of the high-nutrient Pearl River plume may interact with upwelled waters. In addition, it is worthwhile to note that many previous studies on N_2_ fixation were not conducted using the uncontaminated ^15^N_2_ gas dissolution method^27–29^, which introduces great uncertainty when it comes to identifying the favorable environmental conditions for upwelling-associated N_2_ fixation. Therefore, the objectives of this study are to examine whether N_2_ fixation can be important in the DSU and PTU regions using the uncontaminated ^15^N_2_ gas dissolution method, and to explore the potential factors that influence N_2_ fixation in the upwelling regions.

## Results

### Environmental conditions

**Figure 2:**
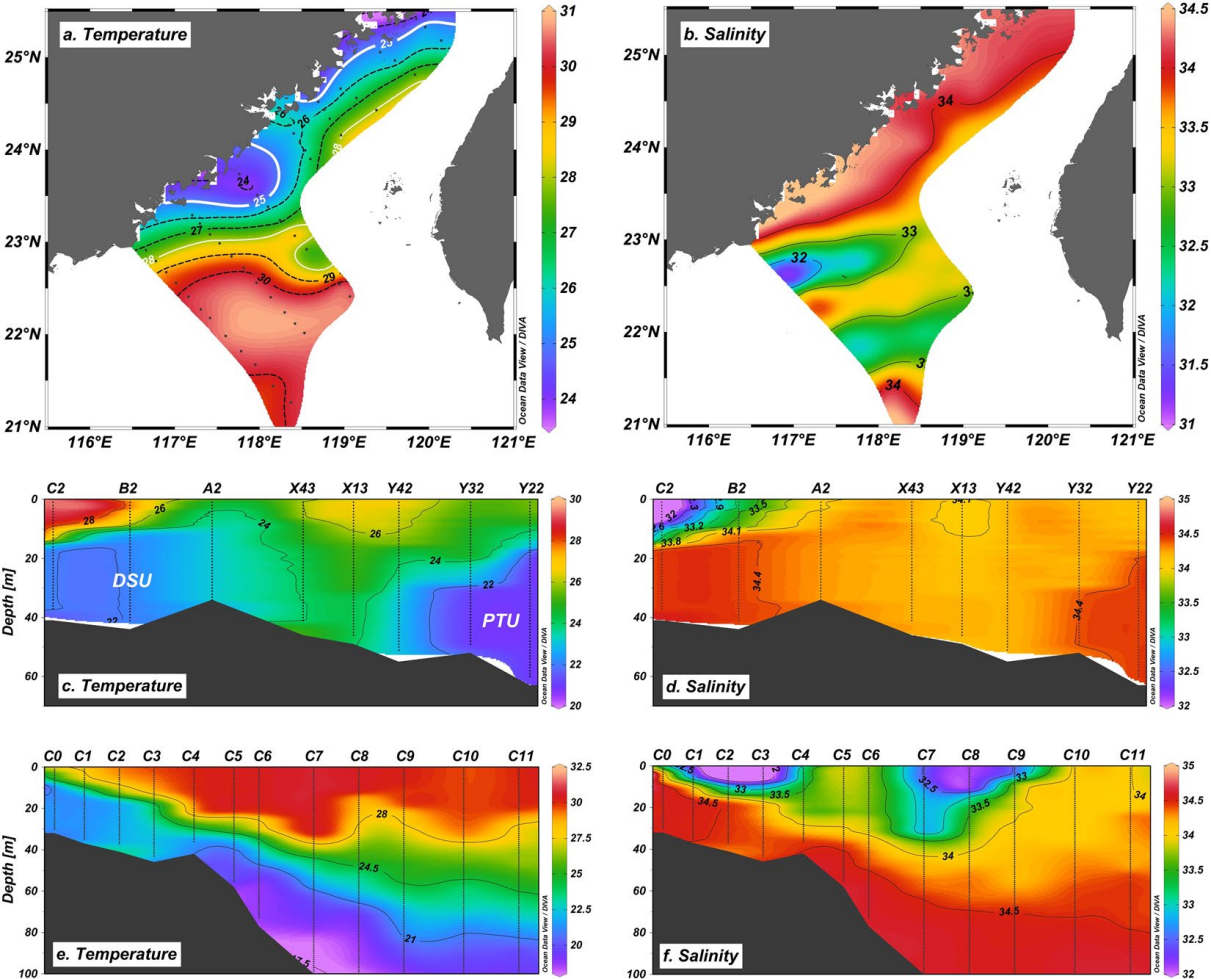
Horizontal and vertical distributions of temperature (°C) and salinity (psu) in the study region. (**a**) Surface temperature, (**b**) surface salinity, and depth profiles of seawater temperature (**c** and **e**) and salinity (**d** and **f**) of the alongshore transect (**c** and **d**), and the C-section (**e** and **f**) as indicated by the light-yellow lines in Fig. 1. This figure was created using Ocean Data View (Schlitzer, R., Ocean Data View, http://odv.awi.de, 2017).

Horizontal distributions of measured seawater temperature and salinity revealed that the sea surface temperature (SST) ranged between 23.9 °C and 30.8 °C (Fig. 2a), and the sea surface salinity (SSS) ranged between 31.2 and 34.4 (Fig. 2b). Water with much lower temperatures (<25 °C) and relatively high salinities (>34.1) was found along the western edge of the TWS, and its distribution was representative of the two main regions previously identified as the DSU and PTU in summer^22^. As revealed by the alongshore transects (Fig. 2c and d), a low temperature and high salinity water column occupied at depths greater than 15 m in both the northern and southern TWS, with relatively high temperature and low salinity water in between at stations X43, X13 and Y42. This indicates that the water masses of the two upwelling regions originate from different water current flows. The Pearl River plume that is characterized by warm (>28.5 °C) and low salinity (<33) water entered the TWS and split into a two-pronged flow pattern (i.e., eastern and the western flows) near stations C5 and C6 (Fig. 2a and b). These two flows may influence waters up to 30 m deep along the western and southern Taiwan Bank (Fig. 2e and f).

**Figure 3:**
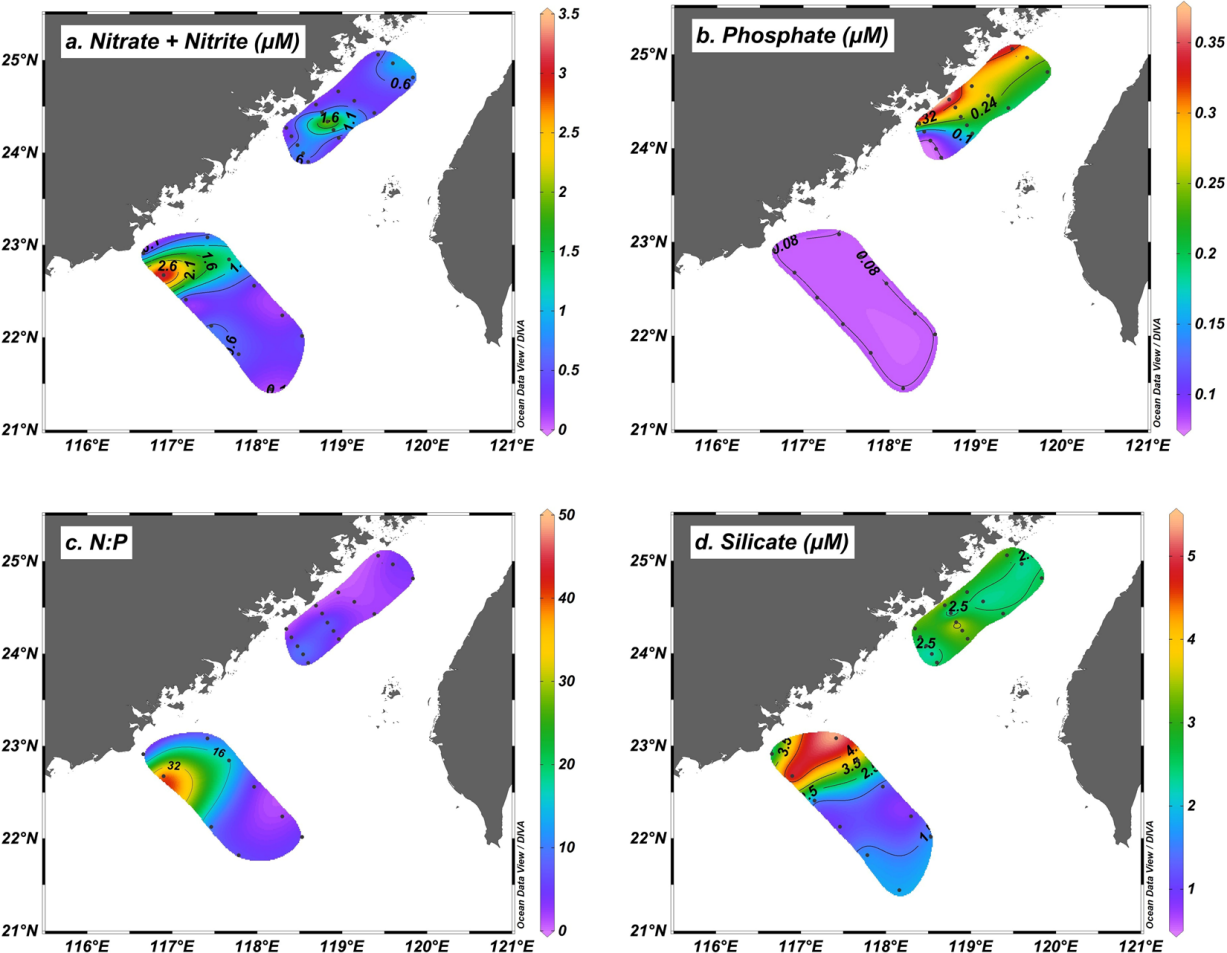
Horizontal distributions of nutrients in the study region. Near-surface concentrations of (**a**) nitrate + nitrite (µM) and (**b**) phosphate (µM), and (**c**) calculated N:P ratios, and (**d**) near-surface concentration of silicate (µM). Note that PO_4_^3−^ concentrations at most stations in the SW area and the oligotrophic region were below the detection limit (0.08 μM). The actual N:P ratios at these stations should therefore be much higher than those calculated based on a PO_4_^3−^ concentration of 0.08 μM (Table S1). This figure was created using Ocean Data View (Schlitzer, R., Ocean Data View, http://odv.awi.de, 2017).

High surface concentrations of nitrate plus nitrite (N + N) (up to 3.49 μM) associated with low salinities (<33) were observed at stations in the southwest area of the DSU region (hereafter the SW area) (Figs 2b, 3a and Table S1), reflecting the predominantly riverine source of N from the Pearl River plume, and N + N concentrations declined dramatically to below 1 μM at stations that were further offshore, toward the oligotrophic region. In contrast, surface N + N was more depleted in the area where the DSU and PTU regions met (hereafter the NE area) (Fig. 3a), with concentrations around or below 1 μM at most stations except X13 (2.59 μM). Surface phosphate (PO_4_^3−^), however, had the opposite distribution pattern to that of N + N, with considerably high concentrations in the NE area (up to 0.37 μM) and values that were below the detection limit at most SW area stations (Fig. 3b). As a result, the calculated N:P ratios of the surface waters were low in the NE area (1–10.4:1) and high in the SW area (e.g., >43.6:1 at station C2) (Fig. 3c). The surface silicate (SiO_4_^2−^) concentration displayed a similar pattern as that of N + N, with high concentrations in the SW area and slightly depleted ones in the NE area (Fig. 3d).

### N_2_ fixation and primary production rates

**Figure 4:**
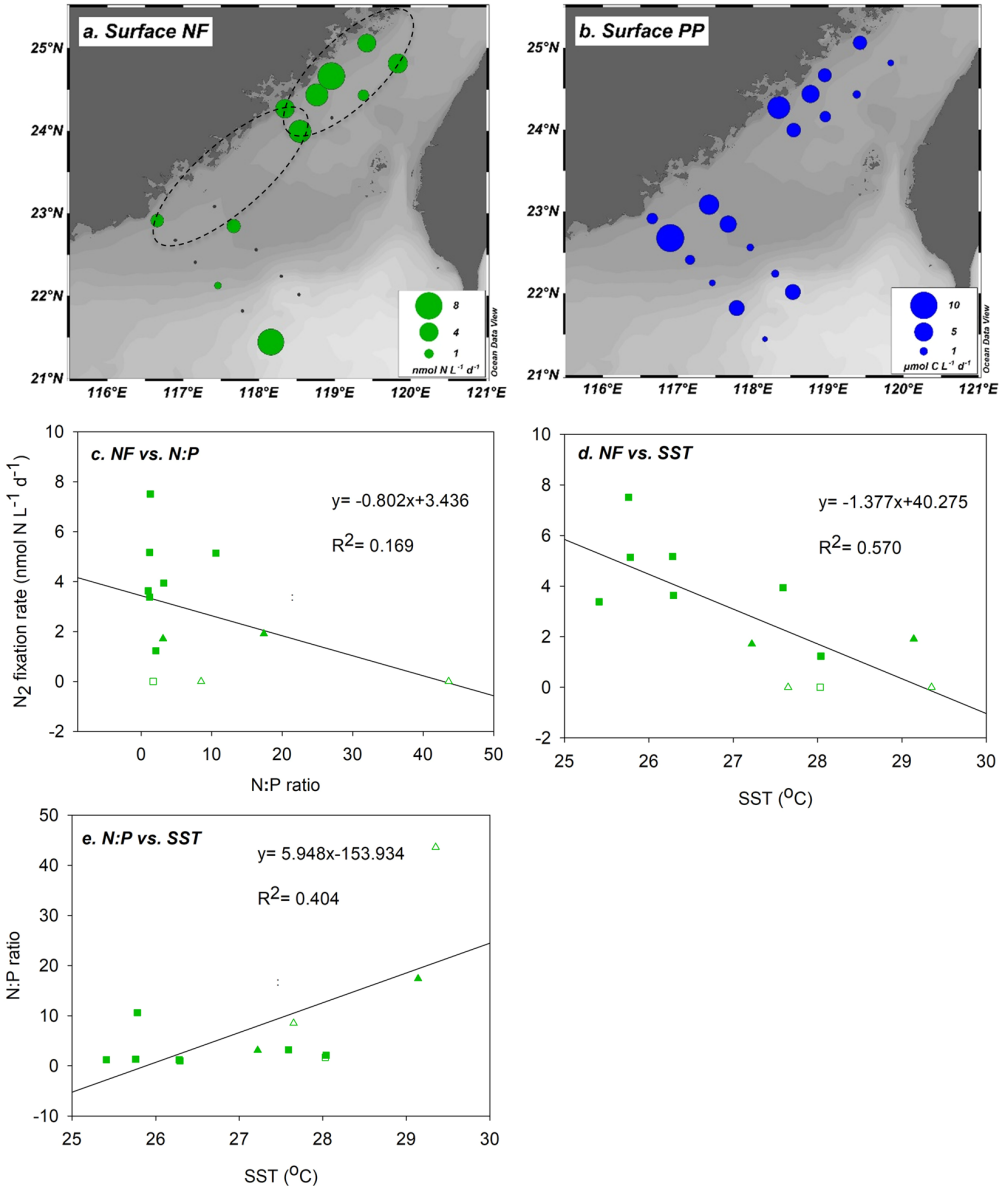
Surface N_2_ fixation (NF) and primary production (PP) rates in the study region. (**a**) NF (nmol N L^−1^ d^−1^) and (**b**) PP (µmol C L^−1^ d^−1^) rates in the surface waters at each station are indicated by circles, with size being proportional to the rate measured. Correlations (**c**) between NF rate and the calculated N:P ratio, (**d**) between NF rate and sea surface temperature (SST, °C), and (**e**) between the N:P ratio and SST in the surface waters of the upwelling regions. The dashed ellipses in (**a**) indicate the Dongshan Upwelling (DSU) and Pingtan Upwelling (PTU) regions. The black dots in (**a**) and the open symbols in (**c**), (**d**), and (**e**) represent N_2_ fixation rates that were below the detection limit (Table S1). Squares and triangles in (**c**), (**d**), and (**e**) represent stations in the NE and SW areas, respectively. NF and PP rates (mean ± SD, n = 3) are shown in Table S1 and were analyzed using SigmaPlot 12.5 (Systat Software Inc.) to test for significant differences between the NE and SW areas, and between coastal waters and the oligotrophic area using t-tests. A significance level of *p* < 0.05 was applied. (**a**) and (**b**) were created using Ocean Data View (Schlitzer, R., Ocean Data View, http://odv.awi.de, 2017).

Surface N_2_ fixation rates ranged from below the detection limit to 7.51 nmol N L^−1^ d^−1^ in our study (Fig. 4a and Table S1). Most of the higher rates occurred in the NE area, while in the SW area, despite not being significant, rates were much lower or even undetectable (<1.91 nmol N L^−1^ d^−1^, *p* = 0.067). Offshore station C10 located towards the oligotrophic region also had high diazotrophic activity, with a N_2_ fixation rate of 7.13 nmol N L^−1^ d^−1^.

Surface primary production ranged from 0.37 to 9.97 μmol C L^−1^ d^−1^ (on average 3.13 ± 2.74 μmol C L^−1^ d^−1^) across the entire study region (Fig. 4b and Table S1). Much higher (although not statistically significant, *p* = 0.072) production rates were observed in the coastal waters than in the oligotrophic area, with the highest rates observed at station C2, which was affected by the western flow of the Pearl River plume (Figs 2b and 4b). Primary production generally decreased seaward, except at stations C8 and B10 in the southern TWS, which were significantly influenced by the eastern flow of the Pearl River plume (Figs 2b and 4b).

### *nifH* gene abundance

**Figure 5:**
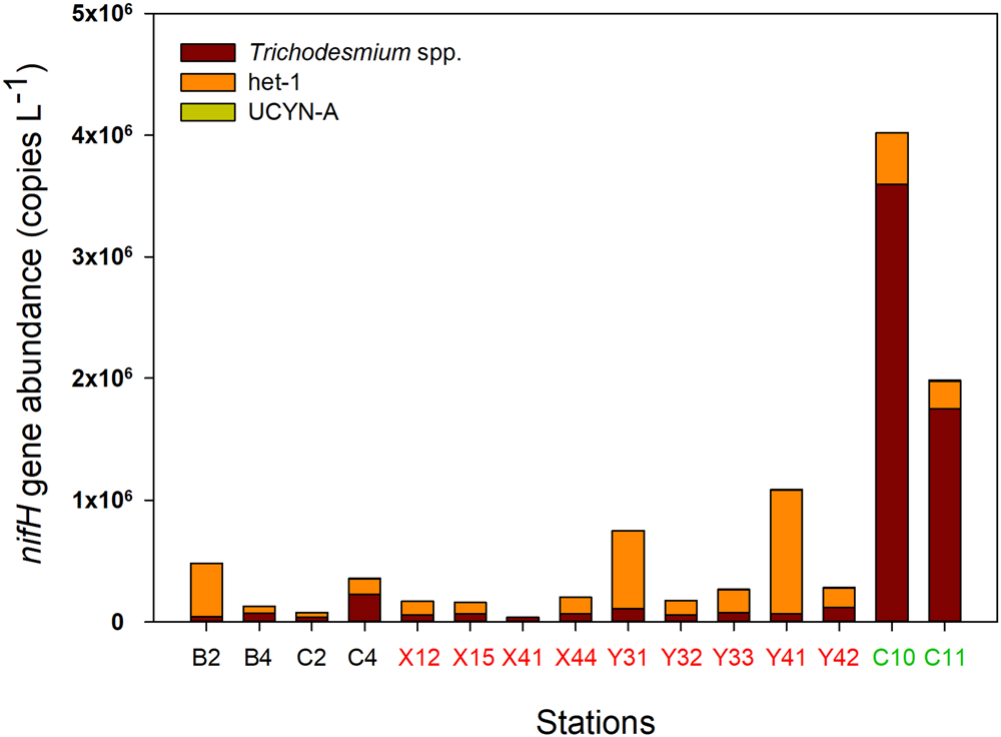
*nifH* gene abundance (copies L^−1^) of three diazotrophic groups that were detected in the study region. Stations in the SW and NE areas of the upwelling regions are denoted by name in black and red, respectively, and those in the oligotrophic region are denoted by name in green. Note that UCYN-A was detected at low abundance (<5.5 × 10^3^ copies L^−1^) that is not visible in the figure, and het-2, het-3, and UCYN-B were all below the detection limits.

qPCR analysis targeted six different *nifH* phylotypes [i.e., *Trichodesmium* spp., *Richelia* associated with *Rhizosolenia* and *Hemiaulus* (het-1 and het-2, respectively), *Calothrix* symbionts of *Chaetoceros* (het-3), and unicellular cyanobacteria groups A and B (UCYN-A and UCYN-B, respectively)]. Results showed that overall het-1 dominated the other diazotrophic groups surveyed at stations within the coastal upwelling regions (up to 10^6^ copies L^−1^), with abundance in the NE and SW areas being generally higher than in the oligotrophic area (*p* = 0.032 and 0.105, respectively) (Fig. 5). In contrast, *Trichodesmium* spp. dominated at the oligotrophic station C10, and on average were an order of magnitude greater than het-1. Although UCYN-A was detectable at most of the stations, this group was present in relatively low abundance (<5.5 × 10^3^ copies L^−1^) (Fig. 5). UCYN-B, het-2 and het-3 were not detected at all stations.

## Discussion

Regions of coastal upwelling driven by the southwesterly monsoon in the TWS have been well defined previously^17,22–25^. In summer, low temperature (<23 °C) and high nutrient (on average 3.27 μM N + N and 0.35 μM PO_4_^3−^) water upwells into the surface layer along the western coast of the TWS^30^, supporting local high productivity^31,32^. In addition, the newly upwelled nutrients can be carried to the East China Sea by the rapid northward transport^33–35^. Such high nutrient, relatively low temperature, and potentially strong turbulent environments are generally considered to be unfavorable for N_2_ fixation^36–38^. In the present study, however, we found that N_2_ fixation can be pronounced in the PTU and DSU regions, especially at stations in the NE area (3.38–7.51 nmol N L^−1^ d^−1^ at temperatures  27.5 °C) (Fig. 4a and Table S1). Despite the possible influence of using different ^15^N_2_ tracer methods for measuring N_2_ fixation rates across studies^27–29^, the rates we observed were several times higher than those in the nearby basin region of the northern South China Sea^39,40^, an oligotrophic area with environmental conditions that are assumed to be more favorable for N_2_ fixation. Our observations also fell within the high-end range of those previously reported for upwelling regions, such as the Vietnam, Peruvian, Californian and the Benguelan upwelling regions^6,8,11,12^.

In our study, the NE area of the upwelling regions where high N_2_ fixation rates were observed generally had low N + N concentrations (<1 µM) with relatively high PO_4_^3−^ levels (up to 0.37 μM) and therefore low N:P ratios (1.0–10.4:1) in the surface waters (Figs 3, 4a and Table S1), which clearly indicated the presence of a N deficiency. However, the N_2_ fixation rate did not seem to be strongly correlated with the N:P ratio (Fig. 4c); rather, we found a robust relationship between the N_2_ fixation rate and sea surface temperature (Fig. 4d). On a global scale, sea surface temperature has been shown to positively affect N_2_ fixation^41^, which may be attributed to a direct effect of temperature on the physiology of diazotrophs^42^, or an indirect temperature influence on other oceanographic features (e.g., irradiation, mixed layer depth, and nutrient conditions)^39–41,43^. In our study, however, the N_2_ fixation rate was found to be negatively correlated with temperature (Fig. 4d). Given the observed positive correlation between temperature and the N:P ratio (Fig. 4e), we suggested that low N:P ratios in the relatively cold upwelled water should have been partially, if not primarily, responsible for the high N_2_ fixation rates observed at low temperatures, as suggested by Gruber, *et al*.^44,45^. Future studies are needed to fully understand how low temperatures may have directly or indirectly affected the diazotrophs, in particular the most abundant het-1, consequently stimulating N_2_ fixation in the upwelling regions of our study.

Previous studies have reported rather low N:P ratios (~4.5:1) in the water column in the middle of the TWS (Table 1)^34^, including regions that are adjacent to the NE area where low N:P ratios and high N_2_ fixation rates were observed in our study. The major nutrient source for this water is deduced to be bottom water from the Penghu Channel, which is diverted into the Wuchou Depression by the blockage of the Zhangyun Ridge (Fig. 1)^34^. As shown previously, upwelling regions (e.g., the Peruvian, Californian and Benguelan upwelling regions) where upwelled water is depleted in N relative to P are often intimately associated with denitrification beneath^6,8,11^. Because the water column in the upwelling regions for our study was not deficient in oxygen (>4 mg L^−1^, data not shown), local water column denitrification can be ruled out. We therefore surmised that the excessive PO_4_^3−^ in the water body may have been sourced from widespread N-deficient subsurface water from the Western North Pacific^46–48^, a result of long range transport of the water column denitrification signal from the Eastern Tropical Pacific, as the Kuroshio Current intrusion occurs^20,21,33^. In addition to water column denitrification, local sedimentary denitrification may also contribute to N loss, but more research is needed to identify the extent that it may influence the water body N:P ratio.

**Table 1:** Observed N:P ratios in different water masses in the TWS and surrounding regions. The N:P ratios of three water masses are shown, representing the water mass of the upwelling regions (i.e., DSU and PTU) of the TWS and the source water from the South China Sea and the Kuroshio.

Water masses	N:P ratios	References
*Taiwan Strait water*		
Surface water in the northwestern TWS	1.02–11.3	This study
Water column in the middle of the strait (Spring)	5.2 ± 2.8	Chung, *et al*.^34^
Water column in the middle of the strait (Summer)	4.2 ± 3.2	Chung, *et al*.^34^
Dongshan upwelled water in Summer, 2005	15.7 ± 0.9	Hu, *et al*.^66^
Dongshan upwelled water in Summer, 2006	15.4 ± 0.4	Hu, *et al*.^66^
Dongshan upwelled water in Summer, 2008	15.1 ± 4.1	Hu, *et al*.^66^
*South China Sea water*		
150 to 1000 m at SEATS	~12–14.5	Wu, *et al*.^47^
Intermediate water at SEATS	12.7–14.3	Wong, *et al*.^67^
*Kuroshio subsurface water*	~14.46	Liu, *et al*.^68^

Aside from low NO_3_^−^ and relatively high PO_4_^3−^ concentrations, Fe is also required for supporting significant N_2_ fixation fluxes^49,50^. As in the Benguelan and Californian upwelling systems^8,11^, it is very unlikely that surface dissolved Fe is limiting in the NE area of the upwelling regions in this study, because relatively high concentrations of this essential trace element can be brought up to the surface by upwelled water from Fe-rich sediments in such a shallow continental shelf. Indeed, as reported in a previous study conducted in the TWS^51^, the surface total dissolved Fe concentration in early summer, the same time period as our study, was up to 3 nM in the area where we observed high N_2_ fixation rates. The abundant Fe coupled with N deficiency and relatively excess P should thus promote surface N_2_ fixation in the NE area of the upwelling regions.

At stations in the NE area of the upwelling regions, het-1 was found to dominate the diazotrophic groups that were surveyed, implying that these heterocystous cyanobacterial diatom symbionts had an ecological advantage over the other diazotrophs examined, such as *Trichodesmium* spp. (Fig. 5). It has been shown that diatom-diazotroph associations (DDAs) can displace *Trichodesmium* spp. as the dominant diazotroph in water bodies with excess P and Si, and the rates of N_2_ fixation in DDA blooms can even exceed vertical NO_3_^−^ fluxes^7,14,52^. Excessive P and potentially Si supplied by the upwelled water in the NE area should thus establish an ideal niche for het-1-associated N_2_ fixation. In contrast, in the Benguelan and the Californian upwelling regions, UCYN-A was the dominant species in the water where high N_2_ fixation rates were observed^8,11^, whereas in the Peruvian upwelling region, none of the known diazotrophic cyanobacteria were found in the euphotic zone^6^.

In comparison with the NE area, although it was not statistically significant (*p* = 0.067), much lower N_2_ fixation rates (<1.91nmol N L^−1^ d^−1^) were found at stations in the southern TWS (except C10, which was considered to be an oligotrophic station). In these areas, the surface water was under the influence of the Pearl River plume. Voss, *et al*. reported enhanced surface N_2_ fixation rates at the nutrient-depleted edge of the coastal upwelling region off Vietnam, likely due to the Mekong River plume that may increase the stability of the water column and/or provide micronutrients^12^. Unlike the Vietnam upwelling region, the SW area of our study that was affected by the western flow of the Pearl River plume had much higher N + N (and SiO_4_^2−^) concentrations together with a P deficiency (below the detection limit) and hence a high N:P ratio (e.g., >43.6:1 at station C2) in the surface water (Fig. 3 and Table S1). This condition, therefore, may have inhibited surface N_2_ fixation but stimulated primary production in the area (Fig. 4a and b). In fact, the low N_2_ fixation rates at most stations in the SW area were consistent with prior observations in the same region during summer (<1 nmol N L^−1^ d^−1^ at the surface)^26^, suggesting that low diazotrophic activity likely persists in this area. Further southward, the low concentrations of N + N and SiO_4_^2−^ in the eastern flow of the Pearl River plume were due to mixing with oceanic water and phytoplankton assimilation, as evidenced by the increasing primary production compared to the surrounding water (Fig. 4b). Diazotrophs in this region thus may have been outcompeted by fast-growing phytoplankton (e.g., diatoms)^53–55^.

At station C10, where high diazotrophic activity was observed (Fig. 4a), the surveyed diazotrophic community was dominated by *Trichodesmium* spp., which was markedly different from the scenario in the NE and SW areas of the upwelling regions (Fig. 5). *Trichodesmium* spp. are known to prosper in stratified, open oceans with low turbulence, slow NO_3_^−^ input from deep water, and a sufficient supply of Fe or P^56^. In summer, it would appear that conditions in both the South China Sea and the upstream Kuroshio are advantageous to *Trichodesmium* spp. The diazotrophs, however, proliferate only in Kuroshio due likely to the relatively shallow nitracline, the stability of the water column, or the “island mass effect” triggered by a terrigenous nutrient supply^57,58^. Thus, the high abundance of *Trichodesmium* spp. at station C10 can probably be attributed to advection of the cyanobacteria from Kuroshio Current intrusion^20,21,33^.

In summary, our study demonstrated that N_2_ fixation can be pronounced in the PTU and DSU regions of the TWS, with most high rates found in the NE area. The high N_2_ fixation rates in the NE area coincided with low N:P ratios associated with low temperatures of the upwelled water, presumably as a result of non-local water column denitrification and local sedimentary denitrification, which remains to be studied. DDAs were found to dominate over the other diazotrophic groups surveyed in the upwelling regions where high diazotrophic activity occurred. The biogeochemical significance of DDAs is that they are efficient in organic matter export. Because high N_2_ fixation rates were associated with high primary production in the surface water, as previously observed^14^, the dominance of DDAs may imply a direct relationship between new N input and effective export production. Further investigations are necessary to determine how common such substantial diazotrophic activities, as observed in the NE area in our study, are in coastal upwelling regions that are traditionally deemed unfavorable for N_2_ fixation. If similar phenomena are widespread, our knowledge of the global marine N-budget may need to be reevaluated.

## Methods

Observations and experiments were conducted on board the R/V *Yanping* 2 from June 25^th^ to July 5^th^, 2015 in the TWS (Fig. 1). Hydrographic measurements were collected using a CTD (Seabird SEB 17 Plus) profiler equipped with a Rosette sampler. Near-surface water (at a depth of 5 m) was sampled using a Teflon diaphragm pump connected to plastic tubing. All materials in contact with the water were acid-washed before use.

### N_2_ fixation and primary production measurement

Near-surface water (1 or 2 L) was collected for estimating the natural ^15^N and ^13^C abundance of particulate organic nitrogen and carbon. Samples were filtered immediately at the beginning of the incubation period. Determination of N_2_ fixation rates was carried out using the ^15^N_2_ gas dissolution method as described in Mohr, *et al*.^28^. A separate batch of ^15^N_2_-enriched seawater was prepared at each station according to Mohr, *et al*.^28^ and Grosskopf, *et al*.^29^. Briefly, filtered seawater was degassed using the method described in Shiozaki, *et al*.^59^. Then, 5 mL of 98% pure ^15^N_2_ gas (Cambridge Isotope Laboratories) was injected into an acid-cleaned, gas-tight plastic bag containing 500 mL of degassed seawater that was gently tapped until the gas dissolved completely. The percentage of ^15^N_2_ in the ^15^N_2_-enriched seawater was validated using a GasBench-IRMS^60^. Near-surface water was added to triplicate acid-cleaned 2.3-L polycarbonate bottles. Before incubation, 60 mL of seawater was extracted from each bottle, and a NaH^13^CO_3_ (99 atom % ^13^C, Cambridge Isotope Laboratories) solution was added at a final tracer concentration of 100 μM. Subsequently, 60 mL of ^15^N_2_-enriched seawater was added to the incubation bottles, with the enriched water constituting approximately 2.6% of the total sample volume. Each bottle was shaken at least five times. Incubation took place in flow-through deck-board incubators under natural sea-surface irradiance for 24 h. After incubation, the samples were filtered (<100 mm Hg) onto pre-combusted (450 °C, 4 h) 25-mm diameter GF/F membranes for the determination of N_2_ fixation and primary production. After filtration, all GF/F filters were immediately stored at −20 °C.

To estimate the natural and tracer-enriched ^15^N and ^13^C abundance, sample filters were first acid fumed to remove the inorganic carbon and were then measured using a Flash 2000 elemental analyzer coupled to a Thermo Finnigan Delta Plus isotope ratio mass spectrometer. The rates of N_2_ fixation and primary production were calculated according to Mohr, *et al*.^28^ and Hama, *et al*.^61^, respectively. The detection limit for N_2_ fixation rates was estimated following Montoya, *et al*.^62^ by taking 4‰ as the minimum acceptable change in the δ^15^N of particulate nitrogen (i.e., a change of 0.00146 in the ^15^N enrichment of particulate nitrogen) (Table S1).

### DNA extraction and *nifH* gene amplification

Surface seawater was sampled using 4-L, acid-rinsed polycarbonate bottles, and 2 to 4 L were filtered onto 0.22-μm polycarbonate membranes (47-mm diameter; Millipore) under a low vacuum pressure of <100 mmHg. The filters were stored in liquid nitrogen until analysis. To extract the DNA, membranes were cut into pieces under sterile conditions and then placed in tubes containing 800 μL of sucrose lysis buffer (40 mM EDTA, 50 mM Tris-HCl, 0.75 M sucrose) for bead beating using 0.1-mm and 0.5-mm glass beads. The cells were broken using a physical method, agitated for 3 min in a Fast Prep machine (MP Biomedicals, USA) and frozen in liquid nitrogen three times. Lysozyme (5 μl; 100 mg mL^−1^) was then added, and the samples were incubated for 1 h at 37 °C. After incubation, the lysate was transferred into a new 2-mL Eppendorf tube. Proteins were digested by incubation with 1% sodium dodecyl sulfate (SDS) and proteinase K (250 μg mL^−1^) at 55 °C for 2 h and were removed by centrifuge at 12,000 g for 20 min at 4 °C after treatment with equal volumes of phenol: chloroform: isoamyl alcohol (25:24:1) containing 5 M NaCl. As a result, the samples were separated into three layers. The top aqueous layer containing genomic DNA was transferred into a new tube, to which an equal volume of chloroform: isoamyl alcohol (24:1) was added, followed by centrifugation at 12,000 g for 20 min at 4 °C. Genomic DNA was purified by precipitation with 100% isopropanol at −20 °C overnight, followed by washing with 70% ethanol and air-drying. Genomic DNA was then eluted into 50-μL TE buffer and stored at −20 °C.

Quantitative PCR analysis of the diazotrophic community was conducted, targeting on the *nifH* phylotypes of *Trichodesmium* spp., three symbiotic strains (het-1, het-2, and het-3), and UCYN-A and UCYN-B using previously designed primers^63–65^. Probes were 5′-labeled with the fluorescent reporter FAM (6-carboxyfluorescein) and 3′-labeled with TAMRA (6-carboxytetramethylrhodamine) as a quenching dye. The *nifH* standards were obtained by cloning the environmental sequences of previous samples from the South China Sea. The DNA concentrations of *nifH* standards were determined using Quant-iTTM Picogreens® dsDNA Reagent and Kits (Invitrogen) using a Fluoroskan Ascent FL fluorescence microplate reader (Thermo Scientific). Quantitative PCR analysis was carried out as previously described, with slight modifications^63^. qPCR reactions were run in triplicate for each environmental DNA sample and each standard, using the following thermal cycle program: 50 °C for 2 min, 94 °C for 10 min, followed by 49 cycles of 95 °C for 15 s, and 60 °C for 1 min. Standards corresponding to between 5 × 10^1^ and 5 × 10^9^ copies per well were amplified in the same 96-well plate. The copy numbers of the target genes in the environmental samples were calculated from the standard curve. The detection limit of the qPCR reaction corresponded to approximately 50 *nifH* gene copies per PCR reaction, which was equivalent to approximately 625 to 1250 gene copies per L of seawater depending on the volume of seawater sample filtered (i.e., 2 to 4 L).

### Nutrient measurements

Nutrient samples were collected in acid-cleaned 50-mL polypropylene bottles and then frozen immediately at −20 °C. In the laboratory, concentrations of nitrate plus nitrite (N + N), phosphate (PO_4_^3−^) and silicate (SiO_4_^2−^) were measured using a Four-channel Continuous Flow Technicon AA3 Auto-Analyzer (Bran-Lube GmbH). The detection limits for N + N, PO_4_^3−^ and SiO_4_^2−^ were 0.1 µmol L^−1^, 0.08 µmol L^−1^, and 0.08 µmol L^−1^, respectively.

### Statistical analysis

N_2_ fixation rates, primary production rates, and *nifH* gene abundance were analyzed using SigmaPlot 12.5 (Systat Software Inc.) to test for significant differences between study regions using t-tests or one-way ANOVA combined with a Tukey post hoc test. A significance level of *p* < 0.05 was applied.

### Data availability

All data generated or analyzed during this study are included in this published article.

## Electronic supplementary material

Supplementary information
