# Supplementary material for: Nitrogen fixation in two coastal upwelling regions of the Taiwan Strait
Source: Sci Rep. 2017 Dec 14;7:17601. doi: 10.1038/s41598-017-18006-5 (PMC5730613; doi:10.1038/s41598-017-18006-5)
Supplement: Supplementary file 1 — Supplementary information [file 41598_2017_18006_MOESM1_ESM.pdf]

Nitrogen fixation in two coastal upwelling regions of the Taiwan Strait

Zuozhu Wen, Wenfang Lin, Rong Shen, Haizheng Hong, Shuh-Ji Kao, Dalin Shi*

State Key Laboratory of Marine Environmental Science, Xiamen University, Xiamen, Fujian, 361102, P. R. China

*Corresponding author: dshi@xmu.edu.cn

Supplementary Table S1. Sea surface temperature (SST, ℃), sea surface salinity (SSS, psu), surface concentrations of nitrate plus nitrite (N+N, μM), phosphate (PO_4^{3-}, μM) and silicate (SiO_3^{2-}, μM), calculated N:P ratio, surface N_2 fixation rate (NF, nmol N L^{-1} d^{-1}), surface primary production rate (PP, μmol C L^{-1} d^{-1}), and the detection limits of NF rate (DL_{NF}, nmol N L^{-1} d^{-1}) in different areas of the study region. The data of NF and PP rates are mean ± SD (n =3).

**Area**	**Station**	**SST**	**SSS**	**N+N**	**PO_4^{3-}**	**SiO_3^{2-}**	**N:P ratio**	**NF[c]**	**PP**	**DL_{NF}**
The southwest area	B2	27.65	33.48	0.68	BD[a]	5.44	>8.5[b]	BD[a]	5.23±2.25	1.31
	B4	29.14	32.18	1.39	BD[a]	3.71	>17.4[b]	1.91±0.36	3.78±0.21	1.08
	C0	27.22	33.40	0.25	BD[a]	2.35	>3.1[b]	1.71±1.08	1.63±.630	0.74
	C2	29.35	31.24	3.49	BD[a]	5.25	>43.6[b]	BD[a]	9.97±3.54	1.14
	C4	30.28	33.53	BD[a]	BD[a]	1.41		BD[a]	1.26±0.44	0.37
The northeast area	X12	26.28	33.76	0.34	0.28	1.22	1.2	5.17±1.65	4.13±0.31	1.27
	X15	28.03	33.52	0.33	0.19	3.16	1.7	BD[a]	1.61±0.33	0.73
	X41	26.29	34.14	0.33	0.32	3.10	1.0	3.63±1.80	6.55±0.37	1.27
	X44	25.78	34.26	0.85	0.08	2.11	10.4	5.14±1.83	2.67±0.76	0.61
	Y31	25.41	34.19	0.40	0.33	3.08	1.2	3.38±1.57	2.52±0.03	1.04
	Y33	27.59	33.88	0.71	0.22	2.66	3.2	3.94±1.29	0.54±0.03	0.44
	Y41	25.76	34.17	0.36	0.28	2.77	1.3	7.51±1.42	2.37±0.27	0.84
	Y43	28.04	33.75	0.42	0.20	2.62	2.1	1.23±0.40	0.80±0.03	0.50
Oligotrophic	B10	30.69	32.36	0.43	BD[a]	1.66	5.4	BD[a]	3.19±0.09	1.05
	C10	29.73	34.06	BD[a]	BD[a]	1.79		7.13±1.16	0.37±0.04	0.24
Others	C6	30.45	33.72	0.71	BD[a]	1.04	>8.9[b]	0.52±0.42	0.52±0.13	0.35
	C8	30.47	32.13	0.53	BD[a]	1.71	>6.6[b]	BD[a]	3.13±0.09	1.19
	B6	30.32	33.40	0.28	BD[a]	1.27	>3.5[b]	BD[a]	0.70±0.05	0.40
	B8	30.75	33.29	0.15	BD[a]	0.84	>1.9[b]	BD[a]	0.77±0.08	0.41

a. Below detection limit.
b. PO_4^{3-} concentration was below the detection limit (0.08 μM), and thus the actual N:P ratio should be much higher than the calculated ones obtained using a PO_4^{3-} concentration of 0.08 μM.
c. NF and PP rates were analyzed using SigmaPlot 12.5 (Systat Software Inc.) to test for significant differences between the NE and SW areas and between coastal waters and the oligotrophic area, respectively, using t-tests. The results showed that the differences were not statistically significant (p = 0.067 and 0.072, respectively).
